# Association of tooth loss with morbidity and mortality by diabetes status in older adults: a systematic review

**DOI:** 10.1186/s12902-021-00830-6

**Published:** 2021-10-19

**Authors:** Karen Raju, George W. Taylor, Peggy Tahir, Susan Hyde

**Affiliations:** 1 Department of Preventive and Restorative Dental Sciences, Division of Oral Epidemiology and Dental Public Health, School of Dentistry, University of California, 707 Parnassus Avenue, Box 0758, San Francisco, CA 94143-0758 USA; 2 University of California, UCSF Library, 530 Parnassus Ave, San Francisco, CA 94143-0840 USA

**Keywords:** Tooth loss, Functional dentition, Tooth retention, Number of teeth, Edentulism, Diabetes, Morbidity, Mortality, Older adults

## Abstract

**Objective:**

This systematic review assesses the association of tooth loss (TL), as the exposure, with morbidity and mortality by diabetes mellitus (DM) status, as the outcome, in older adults.

**Background:**

Individuals with DM have higher prevalence of severe TL and increased risk of developing morbidities and mortality. No systematic review has evaluated the association between TL with morbidity and mortality by DM status.

**Material and methods:**

Comprehensive searches used multiple publication databases containing reports published between 01/01/2000 and 04/21/2021. Two authors independently evaluated included studies for quality and risk of bias using the Critical Appraisal Skills Programme (CASP) checklist for cohort and Center for Evidence-Based Medicine (CEBM) critical appraisal sheet for cross-sectional studies, while a third author arbitrated decisions to resolve disagreements.

**Results:**

Thirteen studies met the inclusion criteria: eight cross-sectional and five cohort. Qualitative review of the included studies indicated TL is associated with increased incidence and prevalence of DM. TL is also associated with DM-related morbidities including greater prevalence of heart disease, diabetic retinopathy, metabolic syndrome; poorer health-related quality of life; poorer survival of participants with chronic kidney disease; and increased medical expenditure. Overall, the quality of the evidence reviewed was medium, as per the Oxford Centre for Evidence-Based Medicine 2011 Levels of Evidence.

**Conclusions/practical implications:**

This review found significant associations of TL with prevalence and incidence of DM and adverse DM-related outcomes. An interprofessional team-care approach that includes an oral health component could benefit the prevention and management of DM.

## Introduction

The World Health Organization (WHO) defines Healthy Ageing “as the process of developing and maintaining the functional ability that enables wellbeing in older age.” [1] WHO anticipates the number of people over the age of 60 years will double by 2050, and significant societal changes are required to ensure members of the elderly population live healthy lives [2]. Severe tooth loss (TL), defined as having fewer than nine remaining permanent teeth, and edentulism are among the leading ten causes of years lived with disability (YLD) for some high-income countries due to their aging populations [3]. One of the objectives of Healthy People 2030 (OH-05) is to “Reduce the proportion of adults aged 45 and older who have lost all of their natural teeth.” [4].

The United States (U.S.) has wide variation in the percentages of the population aged 65 and older who are edentulous, from a low of 13.9% in Hawaii to a high of 47.9% in West Virginia [5]. While an overall reduction occurred for both partial and total TL among adults aged 65 years and older between 1999 and 2004 and between 2011 and 2016 in the U.S., disparities between low- and higher-income adults persisted [6, 7]. Evidence shows cumulative exposure to dental caries and its sequelae, and severe periodontitis are the major reasons for TL in adults [8–10]. Older adults and people with diabetes mellitus (DM) are at higher risk of TL [11, 12]. Literature supports the bidirectional relationship between DM and periodontitis such that persisting hyperglycemia affects periodontitis, [13, 14] while periodontitis affects glycemic control [15, 16]. As a local chronic inflammatory disease responding to a pathogenic bacterial biofilm with host-derived inflammatory mediators and inflammatory cells contributing to destruction of bone and soft tissues supporting the teeth, as stated previously, severe periodontitis can lead to tooth loss. Evidence suggests periodontitis-associated inflammatory mediators enter the systemic circulation and contribute to an elevated systemic inflammation that leads to insulin resistance and subsequent poorer glycemic control [17]. Hyperglycemia, a consequence of poor glycemic control, contributes to microvascular and macrovascular changes and impaired wound healing in the periodontal tissues. Hyperglycemia also leads to non-enzymatic glycation of proteins and lipids that form glycation end-products (AGEs). These AGEs exacerbate sustained periodontal inflammation, impair periodontal tissue repair and result in additional periodontal tissue destruction and potential tooth loss [18].

Diabetes mellitus is a global health problem affecting 463 million people aged 20–79 years in 2019, projected to rise to 700 million by 2045 [19]. In 2018 estimates were that 26.8% of the U.S. population aged 65 years and older had DM (diagnosed and undiagnosed) [20, 21]. Health expenditures in 2019 associated with DM were 760 billion USD worldwide [19]. Blood glucose levels begin to have an impact on morbidity and mortality even below the diagnostic threshold for DM, [22] and DM, along with higher-than-optimal blood glucose, were responsible for 4.2 million deaths [19] worldwide in 2019, many of which were preventable. A recent Centers for Disease Control and Prevention (CDC) report states the prevalence of severe TL (having fewer than nine remaining teeth) was ≥50% higher for adults with uncontrolled diabetes than for those who did not have the condition [23]. While TL is often considered a proxy measure for periodontitis in adults, conclusive evidence for the mechanism by which TL can contribute to the incidence and prevalence of systemic diseases such as DM is lacking [24–26].

A recent systematic review and meta-analysis suggests the exposure of metabolic syndrome (a cluster of conditions that increases the risk of developing cardiovascular disease (CVD) and type 2 DM) is associated positively with the outcome of TL among adults [27]. Additionally, a dose-response meta-analysis suggests TL was associated with coronary heart disease, [28] stroke risk increments, [28] and susceptibility to all-cause mortality (except for circulatory mortality) [29]. Generally, individuals with type 2 DM are at a higher risk of developing CVD [30, 31] and are 2- to 4-fold more likely to die from some form of CVD [32, 33]. Despite the apparent importance of this topic, to our knowledge, no systematic review has evaluated the association between TL and DM status with TL as the exposure. Therefore, this systematic review poses the following question: Is there an association of TL with morbidity and mortality by DM status in older adults? The purpose of this systematic review will be to summarize the current status of the existing evidence comprising the knowledge-base on this topic.

## Methodology

### Search strategy

This systematic review used the Preferred Reporting Items for Systematic Review and Meta-Analysis (PRISMA) [34] and the Population Exposure Comparison Outcome (PECO) framework to generate the research question and justify the eligibility for study inclusion criteria. Two authors (KR and PT) conducted comprehensive literature searches in PubMed, Embase, CINAHL (search limited to academic journal articles), and Web of Science for articles published from January 1, 2000, until April 21, 2021. The searches included both keywords and index terms (MeSH or Emtree vocabularies), which were specific to the individual database. Terms for tooth loss were combined with terms related to diabetes, morbidity, mortality, and aging. Table 1 lists the full search strategies for all databases used.

**Table 1:** Specific keywords and index terms used in database searches

Database	Keywords and Index Terms
PubMed	(“Tooth Loss”[MeSH] OR “tooth loss” OR “dentition status” OR “functional dentition” OR “tooth retention” OR “number of teeth” OR edentulism OR “functional units”) AND (“Diabetes Mellitus/mortality”[MeSH] OR “glycemic control” OR diabetes OR (diabetes AND (mortality OR morbidity))) AND (“Aged”[MeSH] OR aged OR aging OR elder OR elderly OR geriatric OR “older adult” OR “dependent older”)
Web of Science	(“tooth loss” OR “dentition status” OR “functional dentition” OR “tooth retention” OR “number of teeth” OR edentulism OR “functional units”) AND (“glycemic control” OR diabetes OR (diabetes AND (mortality OR morbidity)) AND (aged OR aging OR elder OR elderly OR geriatric OR “older adult” OR “dependent older”)
Embase	(‘tooth loss’/exp. OR ‘tooth loss’ OR ‘dentition status’ OR ‘functional dentition’ OR ‘tooth retention’ OR ‘number of teeth’ OR ‘edentulism’/exp. OR edentulism OR ‘functional units’) AND (‘diabetes mellitus’/exp. OR ‘glycemic control’/exp. OR (diabetes AND (‘mortality’/exp. OR ‘mortality’))) AND (‘aged’/exp. OR aged OR ‘aging’/exp. OR aging OR elder OR ‘elderly’/exp. OR elderly OR ‘geriatric’/exp. OR geriatric OR ‘older adult’/exp. OR ‘older adult’ OR ‘dependent older’)
CINAHL	(“tooth loss” OR “dentition status” OR “functional dentition” OR “tooth retention” OR “number of teeth” OR edentulism OR “functional units”) AND (“glycemic control” OR diabetes OR (diabetes AND (mortality OR morbidity)) AND (aged OR aging OR elder OR elderly OR geriatric OR “older adult” OR “dependent older”)

### Inclusion and exclusion criteria

The inclusion criteria were studies designed as cross-sectional, case-control, cohort, and controlled trials in the English language, that assessed the association between TL (as the exposure) and morbidity and mortality by DM status (as the outcomes) in adults aged 50 years or older. While the literature arbitrarily defines the age criterion for older adults with 60 or 65 years old as the cutoff, this review extended the age criterion to maximize the number of studies included.

The exclusion criteria were studies conducted in animals, case reports, comments on articles, narrative reviews, and abstracts published in journal supplements; studies conducted in people younger than 50 years; studies in which the investigators did not assess the association between TL and morbidity and/or mortality by DM status; studies in which the investigators did not evaluate the exposure (TL) or outcome of interest (morbidity or mortality related to DM); and studies lacking data on TL and DM.

### Data extraction

To identify and sort studies according to the eligibility criteria, the authors used the systematic review tool Rayyan [35]. For calibration, three authors (KR, SH, and GT) individually screened 100 randomly chosen abstracts (using a random number generator [36]). The three authors discussed which abstracts to include, exclude, and any disagreements until achieving consensus. Author KR subsequently reviewed all 1089 abstracts; 930 were excluded, 104 included, and 55 had decisions deferred until further discussion. The three authors reviewed the included and deferred-decision abstracts to reach consensus, resulting in a full manuscript review for 38 shortlisted studies. Data extracted from all studies included: study characteristics (authors, year of publication, country, setting, study design, follow-up period for cohort studies); population characteristics (sample size, sampling method, participants’ age); measurement criteria (determination of number of teeth, diagnostic criteria for DM-associated outcomes); confounders; and results (statistical significance criterion, confidence interval, main conclusions).

### Quality assessment

Two authors (KR and SH) reviewed and appraised all included studies using the Critical Appraisal Skills Programme (CASP) Checklist for Cohort Studies [37] and Center for Evidence-Based Medicine (CEBM) Critical Appraisal of a Cross-Sectional Study (Survey) [38]. The author GT arbitrated decisions where there was disagreement in the full-text reviews. The Oxford Centre for Evidence-Based Medicine 2011 Levels of Evidence table was used to assess the quality of evidence [39].

### Meta-analysis

Measurement heterogeneity for the tooth loss exposure and diabetes/diabetes-related outcomes was not conducive for performing a meta-analysis.

## Results

### Results of the search

**Fig. 1:**
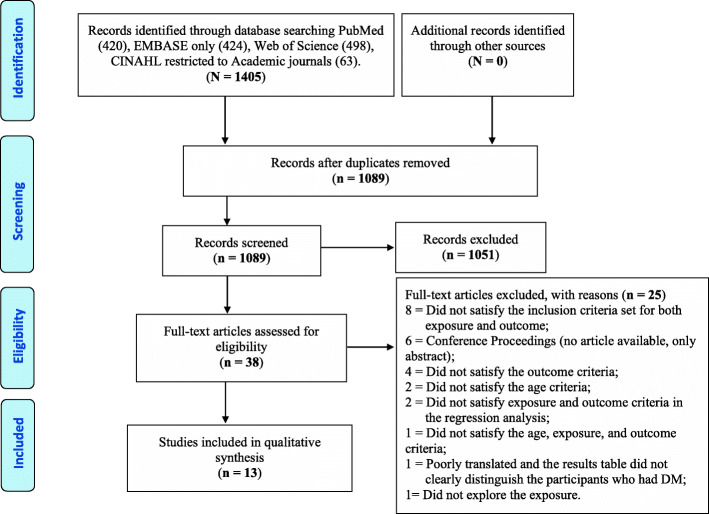
PRISMA flow diagram for selecting studies for inclusion in systematic review

The initial search identified 1405 articles - 420 papers from PubMed, 498 from Web of Science, 424 from Embase, and 63 from CINAHL databases. The Embase search yielded 1860 articles; however, Embase includes both original Embase content and PubMed content; therefore, PubMed references imported into Embase were eliminated before exporting the Embase-only references. After removing duplicates using Zotero 5.0.83 version, [40] 1102 articles remained, and Rayyan [41] identified a further 13 duplicates. A review of the remaining 1089 titles and abstracts yielded 38 potentially eligible studies. The Figure presents the PRISMA flow diagram and describes the process for study selection with the reasons for the exclusion of 25 of 38 potentially eligible studies Fig. 1.

### Description of the included studies

All 13 included articles were observational studies (Tables 2 and 3). Of these, 8 were cross-sectional, and 5 were cohort designs. Three studies were conducted in the U.S.A. [44, 46, 53], 3 in Korea [48–50], 3 in Japan [42, 47, 52], 2 in Finland [24, 43], 1 in Norway [45], and 1 using global data [51].

**Table 2:** Summary of included cohort studies

First Author, Year, Country	Follow -Up Period	N	Age at Baseline, Years	Outcome	Number of Teeth Designation	Results (95% CI)	Summary
					**Remaining Teeth**		
**Suzuki** **[**42] **2021** **Japan**	1 year	1,017,758	50–74	Sum of medical expenditure and pharmacy expenditure	28 or more 25–27 20–24 15–19 10–14 5–9 1–4	Referent group Difference in the medians for public health expenditure in relation to number of teeth was smaller than that for the means.	Patients with DM and a lower number of teeth incurred higher medical expenditure.
**Ruokonen,** **[**43] **2017,** **Finland**	13 years	144	≥ 55^†^ < 55^†^	Mortality due to chronic kidney disease	≤ 25 > 25	Referent group **HR** 0.95 (0.92,0.98)	Participants with chronic kidney disease with > 25 remaining teeth had significantly lower hazard of mortality than those with ≤ 25 remaining teeth, after controlling for diabetic nephropathy (n = 52) vs other types of chronic kidney disease (n = 92) and age.
**Demmer,** **[**44] **2008,** **U.S.A.**	17 ± 4 years	9296	50 ± 19*	Diabetes incidence	24–32 18–23 8–17 1–7 Edentulous	Referent group **OR** NS NS 1.7 ^‡^ (*p* < 0.05) 1.3 (1.00,1.70)	Missing ≥ 25 teeth was significantly associated with increased incidence of diabetes relative to those missing 0–8 teeth. Edentulous participants had 30% greater odds of developing diabetes than periodontally healthy individuals.
					**Missing** **Teeth**		
**Håheim,** **[**45] **2017,** **Norway**	12.5 years	5323	70.4 ± 4.7*	Mortality	0 to 1 2 to 4 5 to 8 9 to 31 Edentulous	Referent group **HR** 1.01 (0.73,1.39) 0.86 (0.57,1.30) 1.14 (0.79,1.66) 1.23 (0.67,2.24)	Number of teeth extracted was not a significant predictor of mortality in men with diabetes.
**Liljestrand,** **[**24] **2015,** **Finland**	13 years	7862	60.8 ± 8.4*	Diabetes incidence	0 to 1 2 to 4 5 to 8 9 to 31 Edentulous	Referent group **HR** 1.20 (0.88,1.63) 1.36 (0.97,1.90) 1.37 (1.02,1.86) 1.56 (1.10,2.20)	Missing ≥ 9 teeth was significantly associated with increased incidence of diabetes.

Abbreviations: N (Number of participants), NS (Nonsignificant), CI (Confidence interval), OR (Odds ratio), HR (Hazard ratio). *Mean ± Standard Deviation age at baseline

^†^ Median age at baseline (years)

^‡^Confidence interval not provided

**Table 3:** Summary of included cross-sectional studies

**First Author, Year, Country**	**N**	**Age**	**Outcome**	**Number of Teeth Designation**	**Results** **(95% CI)**	**Summary**
				**Remaining Teeth**		
**Izuora,** [46] **2019,** **U.S.A.**	301	55.8 ± 11.9*	Heart disease in participants with diabetes	0–28	**OR** 0.960 (0.926,0.998)	Greater number of healthy teeth decreased prevalence of heart disease.
**Itakura,** [47] **2018,** **Japan**	119	86.7 ± 7.8*	Diabetes	0 (0,1.3^†^) + denture; 0 (0,6.5^†^) + no denture	Referent group **OR** 4.45 (1.43,13.88)	Bite instability was significantly associated with increased prevalence of diabetes.
**Shin,** [48] **2017,** **Korea**	3963	60.1–63.6 ± 8.8–9.7*	Metabolic syndrome	28 20–27 0–19	Referent group **OR** 1.45 (1.10,1.91) 1.58 (1.18,2.13)	Missing any teeth was significantly associated with increased prevalence of metabolic syndrome.
**Song,** [49] **2017,** **Korea**	2078	53.0–70.6 ± 0.4–1.1*	Diabetic retinopathy in participants with diabetes	28 20–27 ≤ 19	Referent group **OR** 4.27 (1.38,13.19) 8.73 (2.69,28.33)	Missing any teeth was significantly associated with increased prevalence of diabetic retinopathy.
**Jung,** [50] **2015,** **Korea**	5535	64.9 ± 8.1*	Prediabetes; diabetes	25–32 17–24 1–16 Edentulous	**Diabetes:** Referent group **OR** 1.20 (0.99,1.46) 1.74 (1.35,2.27) 1.72 (1.10,2.70) **Prediabetes:** number of teeth significant for linear trend only (*p* = 0.032)	Missing ≥ 8 teeth was significantly associated with increased prevalence of diabetes but not prediabetes.
**First Author, Year, Country**	**N**	**Age**	**Outcome**	**Number of Teeth Designation**	**Results** **(95% CI)**	**Summary**
				**Remaining Teeth**		
**Vedin,** [51] **2015,** **Global**	15,828	65.0 (59,71)^†^	Diabetes in participants with coronary heart disease	26–32 20–25 15–20 1–14 Edentulous	**OR** 0.89 (0.85,0.92) for each level of tooth loss	Moving from a higher (no teeth) to a lower tooth loss level decreased prevalence of DM by 11%.
**Furukawa,** [52] **2007,** **Japan**	100	59.1 ± 8.4*	Atherogenic factors in participants with diabetes	0–28	Spearman correlation coefficient: −0.275 (p = 0.006) HbA1c; 0.202 (p = 0.048) HDL	HbA1c significantly inversely correlated and HDL cholesterol significantly positively correlated with number remaining teeth in older adults with diabetes.
				**Missing Teeth**		
**Huang,** [53] **2013,** **U.S.A.**	70,363	74.0 ± 0.05*	Health-related quality of life in participants with diabetes	0 Any 1–5 ≥ 6 Edentulous	Referent group **OR** 1.25 (1.13,1.37) 1.08 (0.97,1.20) 1.34 (1.20,1.49) 1.40 (1.25,1.57)	Missing any teeth was significantly associated with HRQOL in older adults with diabetes.

**Abbreviations:** N (Number of participants), NS (Nonsignificant), CI (Confidence interval), OR (Odds ratio), HR (Hazard ratio), HRQOL (Health-related quality of life)

*Mean ± Standard Deviation

^†^Median age (25th, 75th quartile)

### Results of individual studies

Tables 2 and 3 summarize the results of the included cohort and cross-sectional studies, respectively.

### Diabetes incidence and prevalence outcomes

Studies with DM incidence as the outcome concluded missing ≥ 25 teeth [44] (Odds Ratio [OR] 1.7, *p* < 0.05) and ≥ 9 teeth [24] (Hazard Ratio [HR] 1.37; 1.02,1.86) were significantly associated with an increased incidence of DM. Studies with DM prevalence as the outcome showed bite instability, [47] (OR 4.45; 1.43,13.88), and missing ≥ 8 teeth [50] (OR 1.74; 1.35,2.27) were significantly associated with a greater prevalence of DM. One study found a significant linear trend (*p* = 0.032) for TL and the prevalence of prediabetes, [50] but the associations with individual categories for missing teeth were not significant. Another study reported the prevalence of DM decreased by 11% when comparing a higher (no teeth) to a lower tooth loss level (1–14 teeth) [51].

### Diabetes-related outcomes

A study showed HbA1c was inversely correlated (rho − 0.275, *p* = 0.006), while HDL cholesterol was positively correlated (rho 0.202, *p* = 0.048) with the number of remaining teeth in older adults with DM [52]. Included studies indicated TL was associated with greater prevalence of diabetic retinopathy (OR 4.27; 1.38,13.19) [49] and metabolic syndrome (OR 1.45; 1.10,1.91) [48] while greater numbers of healthy teeth was associated with lower prevalence of heart disease in participants both with and without DM (OR 0.960; 0.926,0.998) [46]. One study reported missing any teeth was significantly associated with worse health-related quality of life (HRQOL) in older U.S. adults who had DM (OR 1.25; 1.13,1.37) [53]. Additionally, a study reported participants with chronic kidney disease (CKD) and > 25 remaining teeth had a significantly lower hazard of mortality than those with ≤ 25 remaining teeth, after controlling for diabetic nephropathy (*n* = 52) vs. other types of CKD (*n* = 92) and age [43]. However, another study concluded the number of teeth was not a significant predictor of mortality in men with DM [45]. Patients with DM aged 50–59 years who had 5–9 teeth incurred the highest average public health expenditure while patients with 28 or more teeth incurred low average public health expenditure [52]. However, among patients with DM aged 60–74 years, the more teeth they had the fewer medical expenses incurred except for females aged 70–74 years [42].

### Risks of bias in included studies

Tables 4 and 5 provide a detailed summary of the risk of bias for included cohort and cross-sectional studies using CASP [37] and CEBM [38] quality appraisal approaches.

**Table 4:** Risk of bias for included cohort studies*

First Author, Year, Country	Participant Selection, Sample Size, Response Rate	Measurement	Confounding	Statistical Significance Criterion, Confidence Interval	External Validity/Applicability
**Suzuki** [42] **2021** **Japan**	• National Database of Health Insurance Claims and Specific Health Checkups. • Very large sample size.	**Predictor:** Data extracted for those with periodontitis dental claims which may overestimate TL. Dental linkage performed by hash value from insurer’s ID (ID1), which tends to underestimate TL. Number of teeth included 3rd molars. Not included edentulous. Claims data from multiple dental providers may introduce non-differential misclassification bias for number of teeth. **Outcome:** DM status extracted from medical and pharmacy insurance claims for outpatient services using ICD-10 codes. Data linkage performed by ID1 tends to overestimate patients with DM. Medical expenditure determined by the sum of medical and pharmacy expenditure stored in the national database, recorded in Japanese yen.	**Included:** age and sex. **Not included:** daily oral hygiene, diet, dental care utilization, SES comorbidities, duration of DM, smoking, alcohol use, prescription use of DM medication, cholesterol reducing drugs and antihypertensives, blood pressure, height, weight, waist and hip circumference, cholesterol panel, non-fasting glucose, hs-CRP.	Descriptive statistics. Public Health Expenditure presented as mean and median values with 25th and 75th percentiles.	• National database of Japanese older adults.
**Ruokonen,** [43] **2017, Finland**	• Convenience sample from urban university hospital of patients with CKD at pre-dialysis stage. • No sample size calculation or response rate included	**Predictor:** WHO criteria for oral health status, no information for examiner training or calibration. **Outcome:** national death registry information for 62 of 65 deaths.	**Included:** demographics, smoking, number of medications. **Not included:** daily oral hygiene, diet, dental care utilization, alcohol use, duration of DM, comorbidities.	*P* = 0.10 for entry in multivariable regression model and *P* = 0.05 for removal. HR (95%CI)	• Convenience sample from urban university hospital • Small sample size. • 5% loss-to-follow-up over 13 years. • Finnish study participants may be less diverse than U.S. population.
**Demmer,** [44] **2008,** **U.S.A.**	• National probability sample of non-institutionalized adults. • Large sample size.	**Predictor:** examiners trained for Periodontal Index and DMFT, no kappa scores included. **Outcome:** incident DM identified by death certificate, self-report, pharmacological treatment, hospital stay with discharge diagnosis of DM.	**Included:** demographics, SES, BMI, skin-fold thickness, physical activity, cholesterol, blood pressure, smoking, diet. **Not included:** daily oral hygiene, dental care utilization, alcohol use, duration of DM, prescription use of DM medication, comorbidities.	p ≤ 0.05 OR (95% CI) Multivariable adjustment for potential confounders.	• National probability sample of U.S. non-institutionalized older adults. • 6% loss-to-follow-up over 17 ± 4 years.
**Håheim,** [45] **2017,** **Norway**	• National population-based sample of men only. • Large sample size.	**Predictor:** oral health measures extracted from database. No information on specific indices, examiner training, or calibration. Number teeth included 3rd molars. DM status extracted from database, no criteria for diagnosis included. **Outcome:** national death registry.	**Included:** demographics, smoking, alcohol use, prescription use of DM medication, cholesterol reducing drugs and antihypertensives, blood pressure, height, weight, waist and hip circumference, cholesterol panel, non-fasting glucose, hs-CRP. **Not included:** daily oral hygiene, diet, dental care utilization, comorbidities, duration of DM.	p < 0.05 HR (95%CI) Multivariable adjustment for potential confounders.	• Large, national population-based study limited to men only. • 35% loss to follow-up over 12.5 years. • Norwegian population of men only may be less diverse than U.S. population.
**Liljestrand,** [24] **2015,** **Finland**	• National population-based sample. • Large sample size.	**Predictor:** nurses trained to count teeth but not to distinguish between natural teeth, implants, and pontics therefore bias towards null. No kappa scores. Number teeth included 3rd molars. Survey used WHO’s MONICA protocol. **Outcome:** incident DM determined via national registries of drug reimbursement and hospital discharge.	**Included:** demographics, geographic location, BMI, blood pressure, antihypertensives, physical inactivity, parent with DM, smoking, hs-CRP, diet. **Not included:** daily oral hygiene, dental care utilization, prescription use of DM medication, alcohol use, periodontal disease status at baseline, duration of DM, comorbidities.	p ≤ 0.05 HR (95%CI) Multivariable adjustment for potential confounders.	• Large, national population-based sample. • Loss to follow-up not described. • Finnish population may be less diverse than U.S. population.

**Abbreviations:** CKD (Chronic kidney disease), DMFT (Decayed, Missing, Filled Teeth), DM (Diabetes mellitus), OR (Odds ratio), HR (Hazard ratio), CI (Confidence interval), BMI (Body mass index)

*The criteria of the table extracted from Critical Appraisal Skills Programme (CASP) Checklist for Cohort Studies and Center for Evidence-Based Medicine (CEBM) Critical Appraisal of a Cross-Sectional Study (Survey)

**Table 5:** Risk of bias for included cross-sectional studies*

First Author, Year, Country	Participant Selection, Sample Size, Response Rate	Measurement	Confounding	Statistical Significance Criterion, Confidence Interval	External Validity/Applicability
**Izuora,** [46] **2019,** **U.S.A.**	• Consecutive sample of patients admitted to urban university hospital. • No sample size calculation or response rate included.	**Predictor:** examiners calibrated for number and health of the teeth, no kappa scores reported. **Outcome:** history of stroke or heart disease defined as atherosclerotic heart disease or congestive heart failure. Responses verified through review of medical records.	**Included:** demographics, BMI, smoking, previous hospital admissions, DM history, duration of DM, history of CVD, prescription medication, frequency of tooth brushing, flossing, and using mouthwash. **Not included:** diet, dental care utilization, alcohol use.	p ≤ 0.05 OR (95%CI) Multivariable analysis using linear and logistic regression.	• Consecutive sample from urban university hospital. • Small sample size. • High prevalence of DM (34%) in study participants higher than in U.S. population overall.
**Itakura,** [47] **2018,** **Japan**	• Convenience sample from 2 urban nursing homes. • No sample size calculation or response rate included.	**Predictor:** Eichner index classification of bite stability. Examiner training and calibration not included. **Outcome:** Japan Diabetes Society guidelines for diagnosis of DM.	**Included:** demographics, BMI, serum albumin, blood pressure, chest X-ray, electrocardiogram, dementia, activities of daily living, grip strength, repetitive swallowing, comorbidities. **Not included:** daily oral hygiene, diet, dental care utilization, duration of DM, smoking, alcohol use, prescription use for hypertension, DM, and dyslipidemia.	p < 0.05 OR (95%CI) Multivariable logistic regression analysis.	• Convenience sample from 2 nursing homes may have included healthier participants able to consent and undergo testing. • Small sample size. • 9% of participants excluded due to missing data. • Japanese study participants may be less diverse than U.S. population.
**Shin,** [48] **2017,** **Korea**	• National probability sample of non-institutionalized adults. • Large sample size.	**Predictor:** examiners trained to count teeth, excluded 3rd molars, kappa scores not included. **Outcome:** International Diabetes Federation Task Force on Epidemiology and Prevention guidelines for metabolic syndrome diagnosis.	**Included:** demographics, SES, general health status, oral health status, daily toothbrushing frequency, periodontitis, prescription use for hypertension, DM, and dyslipidemia, smoking, alcohol use, physical activity. **Not included:** dental care utilization, diet, duration of DM, comorbidities.	p < 0.05 OR (95%CI) Multivariable logistic regression analysis.	• National probability sample of noninstitutionalized adults. • 49% of participants excluded due to missing data. • Korean population may be less diverse than U.S. population.
**Song,** [49] **2017,** **Korea**	• National probability sample of non-institutionalized adults. • Large sample size.	**Predictor:** examiners trained to count teeth, excluded 3rd molars, kappa scores not included. **Outcome:** DM diagnosis if fasting blood sugar level was > 126 mg/dL or the participant was currently using antidiabetic medications. ETDRS severity scale for diabetic retinopathy diagnosis.	**Included:** demographics, BMI, waist circumference, blood pressure, smoking, alcohol use, exercise, HbA1c, duration of DM, prescription use of DM medication, frequency of brushing, flossing, mouthwash, interdental brush, electric brush. **Not included:** periodontal disease, diet, dental care utilization.	Two-sided p < 0.05 OR (95%CI) Multivariable logistic regression analysis.	• National probability sample of noninstitutionalized adults. • 20% of participants excluded due to missing data. • Korean population may be less diverse than U.S. population.
**Jung,[**50] **2015,** **Korea**	• District population-based sample of adults aged ≥ 50 years. • Nested cross-sectional study within cohort study.	**Predictor:** periodontal measures reported for pocket depth, gingival recession, bleeding on probing, clinical attachment loss, number of teeth. Trained and calibrated examiners with kappa scores included for periodontal measures. **Outcome:** 2010 American Diabetes Association guidelines for diabetes diagnosis.	**Included:** demographics, blood lipids, blood glucose, HbA1c, BMI, blood pressure, periodontitis, medical history, lifestyle, smoking, year of survey; prescription use for hypertension, DM, and dyslipidemia. **Not included:** daily oral hygiene, diet, dental care utilization, alcohol use, duration of DM, comorbidities.	p < 0.05 OR (95%CI) Multinomial logistic regression analysis.	• District population-based sample of adults aged ≥ 50 years. • 27% response rate. • 1.5% of participants excluded due to missing data. • Korean population may be less diverse than U.S. population.
**Vedin,[**51] **2015,** **Global**	• Nested cross-sectional study of baseline data from global clinical trial of participants aged ≥ 60 years with stable CHD. • Registered with ClinicalTrials.gov.	**Predictor:** number of teeth by self-report, included 3rd molars. **Outcome:** DM which requires pharmacotherapy.	**Included:** demographics, SES, smoking, alcohol use, periodontal disease by self-report of gum bleeding after brushing, physical activity, stress, waist circumference, blood pressure, lipid panel, blood glucose, hs-CRP, WBC, eGFR. **Not included:** daily oral hygiene, diet, prescription use of DM medication, dental care utilization, duration of DM.	p = 0.05 OR (95%CI) Multivariable logistic regression.	• Large global sample of participants aged ≥ 60 years, who have stable CHD, from both developed and developing countries.
**Furukawa,[**52] **2007,** **Japan**	• Convenience sample from 2 urban outpatient diabetes clinics. • No description of inclusion/exclusion criteria. • No sample size calculation or response rate included.	**Predictor:** DMFT excluding 3rd molars, pocket depth, no information provided for examiner training or calibration. **Outcome:** serum lipid panel for atherogenic factors.	**Included:** demographics, BMI, blood pressure, smoking, oral hygiene score, periodontal pocket depth, HbA1c, duration of DM, creatinine, urinalysis. **Not Included:** daily oral hygiene, diet, dental care utilization, alcohol use, prescription use of DM medication, comorbidities.	Two-sided *p*-values Spearman correlation coefficients. Did not report multivariable regression analysis.	• Convenience sample. • Small sample size. • Japanese study participants may be less diverse than U.S. population.
**Huang,[**53] **2013,** **U.S.A.**	• Nationally representative sample of participants aged ≥ 65 years. • Large sample size.	**Predictor:** number of teeth by self-report, included 3rd molars. **Outcome:** health-related quality of life by Healthy Days Core Module.	**Included:** demographics, smoking, dental care utilization, DM by self-report, healthcare utilization. **Not included:** daily oral hygiene, alcohol use, diet, comorbidities, duration of DM, prescription use of DM medication, periodontal disease.	*p* < 0.001 OR (95%CI) Multivariable linear and logistic regression.	• Large nationally representative sample of U.S. population aged ≥ 65 years.

**Abbreviations:** CHD (Coronary heart disease), CKD (Chronic kidney disease), CVD (Cardiovascular disease), DMFT (Decayed, Missing, Filled Teeth), DM (Diabetes mellitus), OR (Odds ratio), HR (Hazard ratio), CI (Confidence interval), BMI (Body mass index), SES (Socio economic status)

*The criteria of the table extracted from Critical Appraisal Skills Programme (CASP) Checklist for Cohort Studies and Center for Evidence-Based Medicine (CEBM) Critical Appraisal of a Cross-Sectional Study (Survey)

Loss to follow-up (LTF) exceeding 20% can pose serious threats to external validity [54]. One study reported 35% LTF over 12.5 years, [45] and 1 study did not report the LTF [24]. Selection bias was a risk for 2 studies that used convenience samples, [47, 52] 1 that used a consecutive sample, [46] 3 that recruited participants through telephone interview, [44, 50, 53] 1 did not describe the participant inclusion/exclusion criteria, [52] and another did not include women [45]. Additionally, measurement bias was present in some studies that identified DM status via self-report, [44] electronic health records, [45] national registries of death, [43–45] drug reimbursement, [24, 42] and hospital discharge diagnosis [24, 44]. Moreover, studies used different categories for the number of missing or remaining teeth, and the denominator for a full dentition varied between 28 and 32 teeth. Five studies, [24, 42, 45, 51, 53] included third molars, which might lead to non-differential misclassification and may result in an underestimate of the strength of association between TL and DM-related outcomes. Further, some studies involved a self-reported number of missing teeth [51, 53] or did not include edentulous participants [42] which also may lead to non-differential misclassification. Many studies mentioned that trained clinicians conducted the oral examinations, but did not report the intra- or inter-rater reliability, [24, 44, 46, 48, 49] while others did not provide any information regarding examiner training or calibration [43, 45, 47, 52]. In 1 study, trained nurses counted the number of missing teeth but were not trained to distinguish natural teeth from, for example, bridge pontics or dental implants, which raised the probability of misclassification bias [24]. Most of the studies did not control for potential confounders such as dental care utilization, daily oral hygiene practices, alcohol use, prescription use of DM medication, and duration of DM.

## Discussion

The studies included in this systematic review indicated a significant association between TL and the incidence [24, 44] and prevalence [50, 51] of DM. Studies showed older adults with DM and substantial TL had significantly greater prevalence of heart disease, [46] diabetic retinopathy, [49] metabolic syndrome, [48] poorer HRQOL [53], and greater medical expenditure [42]. One study found TL was associated with a higher hazard of mortality in participants with CKD [43]. However, another reviewed study found the number of teeth did not significantly predict mortality in men with DM [45]. The strength of the evidence found in this systematic review can be assessed using the Bradford Hill criteria [55] as a framework.

### Biological plausibility

Knowledge is sparse regarding mechanisms directly related to the association of TL with morbidity and mortality by DM status in older adults. In addition to the subsequent host response, oral bacterial colonization can cause chronic inflammation of tooth-supportive tissues, resulting in periodontitis and subsequent TL, [24] as well as more distal, chronic systemic inflammation as indicated by elevated levels of high sensitivity C-reactive protein (hs-CRP) [56]. Hs-CRP measures the general systemic level of inflammation, plays a significant role in diabetogenesis [57–60] and is a strong independent predictor of death in people with DM [61–65]. Moreover, literature proposes that periodontal microbial species and systemic dissemination of bacterial products migrate to distant organs and promote systemic disorders [56, 66, 67]. Additionally, activation of the acute-phase response in the liver and peripheral blood leukocytes results in metastatic periodontal inflammation that causes systemic inflammation leading to oxidative stress, a significant determinant of chronic inflammation [56]. However, these proposed disease mechanisms are models rather than well-established pathways.

Tooth loss as a proxy for severe periodontitis might play an epidemiologically confusing role in the evaluation of a systemic disease hypothesis [24–26]. In addition to being the result of long-term severe periodontitis, TL may be caused by extractions due to dental caries, prosthetic or orthodontic treatment reasons, trauma, or other causes [8–10]. Reasons for TL were not recorded during oral examinations for any of the included studies or were determined from self-report, [51, 53] and some examiners were not trained to distinguish between natural teeth and implants or pontics, [24] which introduces misclassification bias. There is no universal agreement for the definition of a functional dentition. One concept considers the number of opposing natural or prosthetic tooth pairs (i.e. functional units), [68] however no minimum number of functional units defined a functional dentition. A more specific definition of a functional dentition involves the presence of 21 or more permanent teeth (excluding third molars), [69] or 20 or more retained teeth [70]. Moreover, the studies in this review used different categories for grouping the number of missing or remaining teeth and the denominator for a full dentition varied between 28 and 32 teeth.

### Biologic gradient

Number of missing teeth measured by an interval or ordinal scale was associated with significantly poorer DM-related outcomes for some studies included in this review, [42, 46, 51–53] while other studies exhibited a biologic gradient between extent of TL and DM-associated outcomes [24, 44]. For some studies a threshold of TL appeared to be needed in order to significantly impact DM-related outcomes [24, 43, 44, 50]. Dose–response curves are often non-linear and can vary in shape from one study to the next depending on the unique characteristics of the given population, individual susceptibility, and synergistic/antagonistic effects of cumulative exposures [55].

### Consistency

All studies included in this review, except one, [45] consistently demonstrated a significant association between TL and DM-related outcomes in older adults. This review’s finding that retained teeth is inversely correlated with HbA1c in older adults with DM [50, 52] is consistent with previous studies reporting people with high HbA1c have poorer periodontal health and fewer teeth than those without DM [12, 71]. This review also bolstered the findings of studies conducted to explore the relationship between TL and metabolic syndrome [72–74]. The findings of this review are consistent with previous evidence indicating a significant association between periodontitis and DM-related complications such as diabetic retinopathy [75, 76]. The findings of an included study in this review regarding DM in participants with coronary heart disease are consistent with a report in the literature that individuals with CVD who were edentulous or with few remaining teeth had a higher prevalence of DM than participants with many teeth [77]. Additionally, this review supports the evidence concluding an association between TL and incidence of CVD among individuals with DM [78, 79]. The study included in this review regarding the association between TL and HRQOL in older adults with DM, [53] is supported by previous evidence reporting individuals with DM with fewer remaining natural teeth scored lower in physical functioning and role functioning than did those with more retained natural teeth [80]. One included study [42] is the first to report the population-level association between number of teeth and public health expenditure in patients with DM, and supports the evidence of increasing medical costs associated with DM among those aged 65 years and older by 26% from 2012 to 2017 in the U.S. which contributes to a growing economic cost to the Medicare program [81].

### Strength of the association

This review revealed a modest magnitude of association between TL and DM or DM-related outcomes in studies assessing greater TL (OR 1.23–1.74) [24, 44, 45, 48, 50, 53] and 1 study that assessed greater tooth retention (OR 0.89) [51]. Two studies reported medium-to-large associations between TL and DM prevalence (OR 4.45) [47] and diabetic retinopathy (OR 4.27–8.73) [49]. Although the modest associations were statistically significant and adjusted for some confounders, residual confounding could have influenced the relationship between TL and DM-related complications [55]. Diet is an example of a potential confounder that affects both TL [82] and type 2 DM [83] but was not controlled for in most of the studies reviewed. The most common missing confounders in the studies were dental care utilization, daily oral hygiene practices, socioeconomic status (SES), alcohol use, prescription use of DM medication, and duration of DM.

As mentioned previously, criteria varied for the clinical diagnosis of DM [42, 47, 49, 50]. The studies differential use of exposure and outcome measurements could have influenced the differences in strength of association. Further, the onset and symptoms of DM occur gradually, [44] yet only 3 included studies adjusted for duration of DM, [46, 49, 52] and 1 cohort study restricted the analysis to incident DM occurring > 9 years after baseline [44]. Therefore, the duration of DM should be considered in future studies to determine whether the association between TL and DM-related outcomes is confounded or modified by the duration of DM.

### Temporality and experimental manipulation

Despite evidence supporting the bidirectional relationship between DM and periodontitis such that persisting hyperglycemia affects periodontitis, [13, 14] while periodontitis affects glycemic control, [15, 16] this current review indicates relatively little is known regarding the association between TL and DM in older adults. This systematic review found very few studies that met the inclusion criteria, the majority of which were cross-sectional [46–53] and therefore unable to determine temporality of the TL exposure and DM outcome. Similarly, no experimental studies have included minimizing tooth loss as a potential intervention for the prevention and/or treatment of DM.

### Limitation

This review is not part of a registry for systematic reviews and the protocol has not been published.

## Conclusion

This systematic review indicates a significant association in older adults between TL and the incidence and prevalence of DM as well as several DM-related morbidities, mortality, poorer health-related quality of life, and higher medical expenditure. However, caution is necessary when considering these findings due to the paucity of studies addressing this topic and the overall medium level of the quality of the evidence [39] due to the limitations in level of evidence (Levels 1 and 3) and observational designs of all studies reviewed. Applying the Bradford Hill criteria to the findings of this systematic review demonstrated biological plausibility and consistency for the association between TL and DM. The review found a statistically significant but modest level of association between TL and DM-related outcomes, mixed results for a biological gradient of TL associated with DM, limited evidence of temporality for the association, and no evidence from experimental studies. Closing knowledge gaps in the evidence-base regarding associations between TL and DM status in older adults will require high-quality longitudinal studies that use trained and calibrated examiners, differentiate reasons for TL, utilize a standard diagnostic criterion for DM, and record the duration and management of DM. The addition of oral health assessment and/or interventions to clinical trials of prevention and treatment of DM could also strengthen the evidence for an association between TL and DM outcomes.

## Practical implications

The association of TL with the prevalence and incidence of DM and adverse DM-related outcomes, as well as the role of periodontitis as a major cause of TL and contributor to chronic systemic inflammation calls for greater emphasis on an interprofessional team-care approach to dental and medical care of patients with DM. Increasing the awareness of dentists and primary care providers about the possible role of tooth loss with poorer health outcomes should be prioritized. In addition to discipline-specific preventive and therapeutic interactions with patients who have DM, dental care providers should include broader DM-related medical-care and self-care messaging in their patient assessments and education, and medical care providers should include oral health and dental care-related messaging during their patient encounters. The CDC’s National Diabetes Education Program provides informative resources that support the concept of an interprofessional team-care approach to lower the risk for DM-related morbidity and mortality in people with DM [84, 85].

## Data Availability

The study used no quantitative dataset for analysis.

## Appendix

**Table 6:** List of excluded studies

S.NO.	REFERENCE	REASONS FOR EXCLUSION
1.	Kowall B, Holtfreter B, Völzke H, Schipf S, Mundt T, Rathmann W, et al. Pre-diabetes and well-controlled diabetes are not associated with periodontal disease: the SHIP Trend Study. J Clin Periodontol 2015;42:422–30. 10.1111/jcpe.12391.	Did not satisfy the inclusion criteria set for both exposure and outcome
2.	2nd World Congress of Health Research: Viseu – Portugal, 7–8 October 2014. Aten Primaria 2014;46:ii. 10.1016/S0212-6567(14)70066-6.	Conference Proceedings (no article available, only abstract)
3.	Zielinski MB, Fedele D, Forman LJ, Pomerantz SC. Oral health in the elderly with non-insulin-dependent diabetes mellitus. Spec Care Dent Off Publ Am Assoc Hosp Dent Acad Dent Handicap Am Soc Geriatr Dent 2002;22:94–8. 10.1111/j.1754-4505.2002.tb01169.x.	Did not satisfy the inclusion criteria set for both exposure and outcome
4.	Aoyama N, Suzuki J-I, Kobayashi N, Hanatani T, Ashigaki N, Yoshida A, et al. Increased Oral *P. gingivalis* Prevalence in Cardiovascular Patients with Uncontrolled Diabetes Mellitus. Int Heart J 2018;59:802–7. 10.1536/ihj.17-480.	Did not satisfy the outcome criteria
5.	Azogui-Levy S, Dray-Spira R, Attal S, Hartemann A, Anagnostou F, Azerad J. Factors associated with oral health-related quality of life in patients with diabetes. Aust Dent J 2018;63:163–9. 10.1111/adj.12577.	Did not explore the exposure
6.	Botero JE, Yepes FL, Roldán N, Castrillón CA, Hincapie JP, Ochoa SP, et al. Tooth and periodontal clinical attachment loss are associated with hyperglycemia in patients with diabetes. J Periodontol 2012;83:1245–50. 10.1902/jop.2012.110681.	Did not satisfy the age criteria
7.	Castillo R, Fields A, Qureshi G, Salciccioli L, Kassotis J, Lazar JM. Relationship between aortic atherosclerosis and dental loss in an inner-city population. Angiology 2009;60:346–50. 10.1177/0003319708319783.	Did not satisfy exposure and outcome criteria in the regression analysis
8.	Hyvarinen K, Salminen A, Salomaa V, Pussinen PJ. Systemic exposure to a common periodontal pathogen and missing teeth are associated with metabolic syndrome. Acta Diabetol 2015;52:179–82. 10.1007/s00592-014-0586-y.	Did not satisfy the outcome criteria
9.	Iwasaki T, Fukuda H, Kitamura M, Kawashita Y, Hayashida H, Furugen R, et al. Association between number of pairs of opposing posterior teeth, metabolic syndrome, and obesity. ODONTOLOGY 2019;107:111–7. 10.1007/s10266-018-0386-x.	Did not satisfy the outcome criteria
10.	Kaur G, Holtfreter B, Rathmann W, Schwahn C, Wallaschofski H, Schipf S, et al. Association between type 1 and type 2 diabetes with periodontal disease and tooth loss (vol 36, pg 765, 2009). J Clin Periodontol 2009;36:1075–1075. 10.1111/j.1600-051X.2009.01483.x.	Did not satisfy the inclusion criteria set for both exposure and outcome
11.	Oliveira EJP, Rocha VFB, Nogueira DA, Pereira AA. Quality of life and oral health among hypertensive and diabetic people in a Brazilian Southeastern city. Cienc Saude Coletiva 2018;23:763–72. 10.1590/1413-81232018233.00752016.	Poorly translated and the results table did not clearly distinguish the participants who had DM
12.	Oluwagbemigun K, Dietrich T, Pischon N, Bergmann M, Boeing H. Association between Number of Teeth and Chronic Systemic Diseases: A Cohort Study Followed for 13 Years. PloS One 2015;10:e0123879. 10.1371/journal.pone.0123879.	Did not satisfy the age criteria
13.	Patel MH, Kumar JV, Moss ME. Diabetes and tooth loss: an analysis of data from the National Health and Nutrition Examination Survey, 2003–2004. J Am Dent Assoc 1939 2013;144:478–85. 10.14219/jada.archive.2013.0149.	Did not satisfy exposure and outcome criteria in the regression analysis
14.	Watanabe Y, Hirano H, Arai H, Morishita S, Ohara Y, Edahiro A, et al. Relationship Between Frailty and Oral Function in Community-Dwelling Elderly Adults. J Am Geriatr Soc 2017;65:66–76. 10.1111/jgs.14355.	Did not satisfy the inclusion criteria set for both exposure and outcome
15.	Maupome G, Gullion CM, White BA, Wyatt CCL, Williams PM. Oral disorders and chronic systemic diseases in very old adults living in institutions. Spec Care Dent Off Publ Am Assoc Hosp Dent Acad Dent Handicap Am Soc Geriatr Dent 2003;23:199–208.	Did not satisfy the inclusion criteria set for both exposure and outcome
16.	Campus G, Salem A, Uzzau S, Baldoni E, Tonolo G. Diabetes and periodontal disease: a case-control study. J Periodontol 2005;76:418–25. 10.1902/jop.2005.76.3.418.	Did not satisfy the age, exposure, and outcome criteria
17.	Aoyama N, Suzuki J-I, Kobayashi N, Hanatani T, Ashigaki N, Yoshida A, et al. Japanese Cardiovascular Disease Patients with Diabetes Mellitus Suffer Increased Tooth Loss in Comparison to Those without Diabetes Mellitus -A Cross-sectional Study. Intern Med Tokyo Jpn 2018;57:777–82. 10.2169/internalmedicine.9578-17.	Did not satisfy the inclusion criteria set for both exposure and outcome
18.	Hess G, Weber D, Kaltheuner M, Molinski M, Scheper N, Reuter HM, et al. Oral health of patients with diabetes in specialized practices in Germany: An ignored issue? Diabetes 2015;64:A690. 10.2337/db1526832798.	Conference Proceedings (no article available, only abstract)
19.	Borges-Yañez SA, Pérez RCC. Oral health related quality of life in diabetics with oral problems. Diabetes 2015;64:A386. 10.2337/db1514721800.	Conference Proceedings (no article available, only abstract)
20.	Al-Emadi A, Bissada N, Farah C, Siegel B, Al-Zaharani M. Systemic diseases among patients with and without alveolar bone loss. Quintessence Int Berl Ger 1985 2006;37:761–5.	Did not satisfy the inclusion criteria set for both exposure and outcome
21.	Thanish Ahamed S, Rajasekar A, Mathew MG. Assessment of tooth loss in chronic periodontitis patients with and without diabetes mellitus: A cross-sectional study. Int J Res Pharm Sci 2020;11:1927–31. 10.26452/ijrps.v11iSPL3.3649.	Did not satisfy the inclusion criteria set for both exposure and outcome
22.	Liljestrand JM, Salminen A, Lahdentausta L, Paju S, Mantyla P, Buhlin K, et al. Association between dental factors and mortality. Int Endod J n.d. 10.1111/iej.13458.	Did not satisfy the outcome criteria
23.	Adam HS, Zhang S, Philips K, Moss K, Wu D, Selvin E, et al. Periodontal disease is associated with risk of incident diabetes among non-obese individuals. Circulation 2020;141. 10.1161/circ.141.suppl_1.P442.	Conference Proceedings (no article available, only abstract)
24.	Wu B, Luo H. DIABETES, POOR ORAL HEALTH, AND COGNITIVE FUNCTION: FINDINGS FROM A NATIONAL SURVEY IN THE U.S. Alzheimers Dement 2019;15:P825–6. 10.1016/j.jalz.2019.06.2946.	Conference Proceedings (no article available, only abstract)
25.	Funakoshi S, Ohata Y, Fujimori K, Etoh R, Sawase K, Hashiguchi J, et al. Number of intact teeth and nutritional status are correlated in hemodialysis (HD) patients with type 2 diabetes. Diabetes 2018;67:A576.	Conference Proceedings (no article available, only abstract)

## Abbreviations

TL: tooth loss
DM: diabetes mellitus
U.S.: United States
CDC: Centers for Disease Control and Prevention
HRQOL: health-related quality of life
CASP: Critical Appraisal Skills Programme
CEBM: Center for Evidence-Based Medicine

## References

[1] WHO | What is Healthy Ageing? n.d. https://www.who.int/ageing/healthy-ageing/en/ (accessed November 25, 2019).

[2] WHO: Number of people over 60 years set to double by 2050; major societal changes required. World Health Organ 2015. https://www.who.int/mediacentre/news/releases/2015/older-persons-day/en/ (accessed March 22, 2020).

[3] Vos T., Abajobir A.A., Abbafati C., Abbas K.M., Abate K.H., Abd-Allah F., et al. Global, regional, and national incidence, prevalence, and years lived with disability for 328 diseases and injuries for 195 countries, 1990-2016: a systematic analysis for the global burden of disease study 2016. Lancet 2017;390:1211–1259. 10.1016/S0140-6736(17)32154-32152, 10100. 10.1016/S0140-6736(17)32154-2 PMC5605509 28919117

[4] National Academies of Sciences, Engineering, and Medicine; Health and Medicine Division; Board on Population Health and Public Health Practice; Committee on Informing the Selection of Health Indicators for Healthy People 2030 (2020). Leading Health Indicators 2030: Advancing Health, Equity, and Well-Being.

[5] Tomar S. Total tooth loss among persons aged greater than or equal to 65 years-selected states, 1995–1997. n.d. https://profiles.nlm.nih.gov/ps/access/NNBBJZ.ocr (, USAaccessed July 19, 2019).

[6] Oral Health Surveillance Report, 2019. Cent Dis Control Prev 2019. https://www.cdc.gov/oralhealth/publications/OHSR-2019-index.html (accessed March 22, 2020).

[7] Griffin SO, Griffin PM, Li C-H, Bailey WD, Brunson D, Jones JA (2019). Changes in older adults’ Oral health and disparities: 1999 to 2004 and 2011 to 2016. J Am Geriatr Soc.

[8] Aida J, Ando Y, Akhter R, Aoyama H, Masui M, Morita M (2006). Reasons for permanent tooth extractions in Japan. J Epidemiol.

[9] van der Velden U, Amaliya A, Loos BG, Timmerman MF, van der Weijden FA, Winkel EG, Abbas F (2015). Java project on periodontal diseases: causes of tooth loss in a cohort of untreated individuals. J Clin Periodontol.

[10] Phipps KR, Stevens VJ (1995). Relative contribution of caries and periodontal disease in adult tooth loss for an HMO dental population. J Public Health Dent.

[11] Helal O, Göstemeyer G, Krois J, Fawzy El Sayed K, Graetz C, Schwendicke F (2019). Predictors for tooth loss in periodontitis patients: systematic review and meta-analysis. J Clin Periodontol.

[12] Patel MH, Kumar JV, Moss ME. Diabetes and tooth loss: an analysis of data from the National Health and Nutrition Examination Survey, 2003-2004. J Am Dent Assoc 1939 2013;144:478–85. 10.14219/jada.archive.2013.0149, 144, 5, 478, 485. 10.14219/jada.archive.2013.0149 23633695

[13] Lamster IB, Lalla E, Borgnakke WS, Taylor GW. The relationship between oral health and diabetes mellitus. J Am Dent Assoc 1939 2008;139 Suppl:19S–24S. 10.14219/jada.archive.2008.0363. 10.14219/jada.archive.2008.0363 18809650

[14] Mealey BL. Periodontal disease and diabetes. A two-way street. J Am Dent Assoc 1939 2006;137 Suppl:26S–31S. 10.14219/jada.archive.2006.0404. 10.14219/jada.archive.2006.0404 17012733

[15] Sanz M, Ceriello A, Buysschaert M, Chapple I, Demmer RT, Graziani F, Herrera D, Jepsen S, Lione L, Madianos P, Mathur M, Montanya E, Shapira L, Tonetti M, Vegh D (2018). Scientific evidence on the links between periodontal diseases and diabetes: consensus report and guidelines of the joint workshop on periodontal diseases and diabetes by the international diabetes federation and the European Federation of Periodontology. Diabetes Res Clin Pract.

[16] Chapple ILC, Genco R (2013). Working group 2 of the joint EFP/AAP workshop. Diabetes and periodontal diseases: consensus report of the joint EFP/AAP workshop on periodontitis and systemic diseases. J Periodontol.

[17] Genco RJ, Graziani F, Hasturk H. Effects of periodontal disease on glycemic control, complications, and incidence of diabetes mellitus. Periodontol 2000 2020;83:59–65. 10.1111/prd.12271. 10.1111/prd.12271 32385875

[18] Borgnakke WS, Genco RJ, Eke PI, Taylor GW. Oral Health and Diabetes. In: Cowie CC, Casagrande SS, Menke A, Cissell MA, Eberhardt MS, Meigs JB, et al., editors. Diabetes Am. 3rd ed., Bethesda (MD): National Institute of Diabetes and Digestive and Kidney Diseases (US); 2018. 33651538

[19] International Diabetes Federation - Facts & figures. Int Diabetes Fed - Home 2019. https://www.idf.org/aboutdiabetes/what-is-diabetes/facts-figures.html (accessed January 30, 2020).

[20] National Diabetes Statistics Report 2020. Estimates of diabetes and its burden in the United States Centers for Disease Control and Prevention; 2020.

[21] Statistics About Diabetes. Stat Diabetes ADA 2018. https://www.diabetes.org/resources/statistics/statistics-about-diabetes (accessed December 28, 2019).

[22] Roglic G, World Health Organization (2016). Global report on diabetes.

[23] Parker ML. Prevalence of and Changes in Tooth Loss Among Adults Aged ≥50 Years with Selected Chronic Conditions — United States, 1999–2004 and 2011–2016. MMWR Morb Mortal Wkly Rep 2020;69. 10.15585/mmwr.mm6921a1, 69, 21, 641, 646. 10.15585/mmwr.mm6921a1 PMC7269607 32463807

[24] Liljestrand JM, Havulinna AS, Paju S, Mannisto S, Salomaa V, Pussinen PJ (2015). Missing teeth predict incident cardiovascular events, diabetes, and death. J Dent Res.

[25] Demmer RT, Desvarieux M (2006). Periodontal infections and cardiovascular disease: the heart of the matter. J Am Dent Assoc.

[26] Desvarieux M, Demmer RT, Rundek T, Boden-Albala B, Jacobs DRJ, Papapanou PN, et al. Relationship between periodontal disease, tooth loss, and carotid artery plaque: the Oral infections and vascular disease epidemiology study (INVEST). Stroke 2003;34:2120–2125. 10.1161/01.STR.0000085086.50957.22, 9. 10.1161/01.STR.0000085086.50957.22 PMC2677013 12893951

[27] Souza ML, Massignan C, Glazer Peres K, Aurélio Peres M. Association between metabolic syndrome and tooth loss: A systematic review and meta-analysis. J Am Dent Assoc 2019;150:1027–1039.e7. 10.1016/j.adaj.2019.07.023. 10.1016/j.adaj.2019.07.023 31761016

[28] Cheng F, Zhang M, Wang Q, Xu H, Dong X, Gao Z, Chen J, Wei Y, Qin F (2018). Tooth loss and risk of cardiovascular disease and stroke: a dose-response meta analysis of prospective cohort studies. PLoS One.

[29] Peng J, Song J, Han J, Chen Z, Yin X, Zhu J, et al. The relationship between tooth loss and mortality from all causes, cardiovascular diseases, and coronary heart disease in the general population: systematic review and dose–response meta-analysis of prospective cohort studies. Biosci Rep. 2019;39(1). 10.1042/BSR20181773. 10.1042/BSR20181773 PMC6328868 30530864

[30] Turner RC, Millns H, Neil HA, Stratton IM, Manley SE, Matthews DR (1998). Risk factors for coronary artery disease in non-insulin dependent diabetes mellitus: United Kingdom prospective diabetes study (UKPDS: 23). BMJ.

[31] Chait A, Bornfeldt KE. Diabetes and atherosclerosis: is there a role for hyperglycemia? J Lipid Res 2009;50 Suppl:S335–339. 10.1194/jlr. R800059-JLR200. 10.1194/jlr.R800059-JLR200 PMC2674740 19029122

[32] Haffner SJ, Cassells H (2003). Hyperglycemia as a cardiovascular risk factor. Am J Med.

[33] Malmberg K, Yusuf S, Gerstein HC, Brown J, Zhao F, Hunt D, Piegas L, Calvin J, Keltai M, Budaj A, Investigators OASISR (2000). Impact of diabetes on long-term prognosis in patients with unstable angina and non-Q-wave myocardial infarction: results of the OASIS (organization to assess strategies for ischemic syndromes) registry. Circulation.

[34] Moher D, Stewart L, Shekelle P (2016). Implementing PRISMA-P: recommendations for prospective authors. Syst Rev.

[35] Ouzzani M, Hammady H, Fedorowicz Z, Elmagarmid A. Rayyan—a web and mobile app for systematic reviews 2016. https://doi.org/DOI: 10.1186/s13643-016-0384-4, 5, 1, 210. 10.1186/s13643-016-0384-4 PMC5139140 27919275

[36] Research Randomizer. Pair dice n.d. https://www.randomizer.org/ (accessed February 5, 2020).

[37] CASP Checklists - CASP - critical appraisal skills Programme. CASP 2018. https://casp-uk.net/casp-tools-checklists/ (accessed January 30, 2020).

[38] Critical Appraisal tools. CEBM 2014. https://www.cebm.net/2014/06/critical-appraisal/ (accessed January 30, 2020).

[39] Howick J, Iain C, Glasziou P, Greenhalgh T, Heneghan C, Liberati A, et al. “The Oxford 2011 Levels of evidence”. Oxford Centre for Evidence-Based Medicine. n.d. https://www.cebm.net/index.aspx?o=5653 (accessed June 6, 2020).

[40] Zotero 5.0.83 version: Editor, under Creator column, problem. Zotero Forums n.d. https://forums.zotero.org/discussion/81610/zotero-5-0-83-version-editor-under-creator-column-problem (accessed 29 Feb, 2020).

[41] Rayyan QCRI, the Systematic Reviews web app n.d. https://rayyan.qcri.org/welcome (accessed February 5, 2020).

[42] Suzuki S, Noda T, Nishioka Y, Myojin T, Kubo S, Imamura T, Kamijo H, Sugihara N (2021). Evaluation of public health expenditure by number of teeth among outpatients with diabetes mellitus. Bull Tokyo Dent Coll.

[43] Ruokonen H, Nylund K, Furuholm J, Meurman JH, Sorsa T, Kotaniemi K, Ortiz F, Heikkinen AM (2017). Oral health and mortality in patients with chronic kidney disease. J Periodontol.

[44] Demmer RT, Jacobs DRJ, Desvarieux M (2008). Periodontal disease and incident type 2 diabetes: results from the first National Health and nutrition examination survey and its epidemiologic follow-up study. Diabetes Care.

[45] Lund Haheim L, Ronningen KS, Enersen M, Olsen I (2017). The predictive role of tooth extractions, Oral infections, and hs-C-reactive protein for mortality in individuals with and without diabetes: a prospective cohort study of a 12 1/2-year follow-up. J Diabetes Res.

[46] Izuora K, Yousif A, Allenback G, Gewelber C, Neubauer M (2019). Relationship between dental loss and health outcomes among hospitalized patients with and without diabetes. J Investig Med.

[47] Itakura S, Miyata M, Kuroda A, Setoguchi M, Kusumoto A, Hokonohara D, Ohishi M (2018). The Association of Bite Instability and Comorbidities in elderly people. Intern Med Tokyo Jpn.

[48] Shin H-S (2017). The number of teeth is inversely associated with metabolic syndrome: a Korean Nationwide population-based study. J Periodontol.

[49] Song SJ, Han K, Lee S-S, Park J-B (2017). Association between the number of natural teeth and diabetic retinopathy among type 2 diabetes mellitus: the Korea national health and nutrition examination survey. Medicine (Baltimore).

[50] Jung Y-S, Shin M-H, Kweon S-S, Lee Y-H, Kim O-J, Kim Y-J, Chung HJ, Kim OS (2015). Periodontal disease associated with blood glucose levels in urban Koreans aged 50 years and older: the Dong-gu study. Gerodontology.

[51] Vedin O, Hagstrom E, Gallup D, Neely ML, Stewart R, Koenig W (2015). Periodontal disease in patients with chronic coronary heart disease: prevalence and association with cardiovascular risk factors. Eur J Prev Cardiol.

[52] Furukawa T, Wakai K, Yamanouchi K, Oshida Y, Miyao M, Watanabe T, Sato Y (2007). Associations of periodontal damage and tooth loss with atherogenic factors among patients with type 2 diabetes mellitus. Intern Med Tokyo Jpn.

[53] Huang DL, Chan KCG, Young BA (2013). Poor oral health and quality of life in older U.S. adults with diabetes mellitus. J Am Geriatr Soc.

[54] Dettori JR (2011). Loss to follow-up. Evid-Based Spine-Care J.

[55] Fedak KM, Bernal A, Capshaw ZA, Gross S (2015). Applying the Bradford Hill criteria in the 21st century: how data integration has changed causal inference in molecular epidemiology. Emerg Themes Epidemiol.

[56] Hasturk H, Kantarci A. Activation and resolution of periodontal inflammation and its systemic impact. Periodontol 2000 2015;69:255–73. 10.1111/prd.12105. 10.1111/prd.12105 PMC4530469 26252412

[57] Engebretson S, Chertog R, Nichols A, Hey-Hadavi J, Celenti R, Grbic J (2007). Plasma levels of tumour necrosis factor-alpha in patients with chronic periodontitis and type 2 diabetes. J Clin Periodontol.

[58] Pradhan AD, Manson JE, Rifai N, Buring JE, Ridker PM (2001). C-reactive protein, interleukin 6, and risk of developing type 2 diabetes mellitus. JAMA.

[59] Wang X, Bao W, Liu J, OuYang Y-Y, Wang D, Rong S, Xiao X, Shan ZL, Zhang Y, Yao P, Liu LG (2013). Inflammatory markers and risk of type 2 diabetes. Diabetes Care.

[60] Hu FB, Meigs JB, Li TY, Rifai N, Manson JE (2004). Inflammatory markers and risk of developing type 2 diabetes in women. Diabetes.

[61] Lund Håheim L, Nafstad P, Olsen I, Schwarze P, Rønningen KS (2009). C-reactive protein variations for different chronic somatic disorders. Scand J Public Health.

[62] Bruno G, Fornengo P, Novelli G, Panero F, Perotto M, Segre O, Zucco C, Deambrogio P, Bargero G, Perin PC (2009). C-reactive protein and 5-year survival in type 2 diabetes: the Casale Monferrato study. Diabetes.

[63] Linnemann B, Voigt W, Nobel W, Janka HU (2006). C-reactive protein is a strong independent predictor of death in type 2 diabetes: association with multiple facets of the metabolic syndrome. Exp Clin Endocrinol Diabetes Off J Ger Soc Endocrinol Ger Diabetes Assoc.

[64] Belalcazar LM, Reboussin DM, Haffner SM, Hoogeveen RC, Kriska AM, Schwenke DC, Tracy RP, Pi-Sunyer FX, Ballantyne CM, for the Look AHEAD Research Group (2010). A 1-year lifestyle intervention for weight loss in individuals with type 2 diabetes reduces high C-reactive protein levels and identifies metabolic predictors of change: from the look AHEAD (action for health in diabetes) study. Diabetes Care.

[65] Landman GWD, Kleefstra N, Groenier KH, Bakker SJL, Groeneveld GH, Bilo HJG, van Hateren KJJ (2016). Inflammation biomarkers and mortality prediction in patients with type 2 diabetes (ZODIAC-27). Atherosclerosis.

[66] Pussinen PJ, Havulinna AS, Lehto M, Sundvall J, Salomaa V (2011). Endotoxemia is associated with an increased risk of incident diabetes. Diabetes Care.

[67] Pussinen PJ, Tuomisto K, Jousilahti P, Havulinna AS, Sundvall J, Salomaa V (2007). Endotoxemia, immune response to periodontal pathogens, and systemic inflammation associate with incident cardiovascular disease events. Arterioscler Thromb Vasc Biol.

[68] Hildebrandt GH, Loesche WJ, Lin CF, Bretz WA. Comparison of the number and type of dental functional units in geriatric populations with diverse medical backgrounds. J Prosthet Dent 1995;73:253–261. 10.1016/s0022-3913(05)80202-1, 3. 10.1016/s0022-3913(05)80202-1 7760274

[69] Dye BA, Weatherspoon DJ, Mitnik GL. Tooth loss among older adults according to poverty status in the United States from 1999 through 2004 and 2009 through 2014. J Am Dent Assoc 2019;150:9–23.e3. 10.1016/j.adaj.2018.09.010. 10.1016/j.adaj.2018.09.010 PMC6394416 30503018

[70] Yamanaka K, Nakagaki H, Morita I, Suzaki H, Hashimoto M, Sakai T (2008). Comparison of the health condition between the 8020 achievers and the 8020 non-achievers. Int Dent J.

[71] Costa FO, Miranda Cota LO, Pereira Lages EJ, Soares Dutra Oliveira AM, Dutra Oliveira PA, Cyrino RM, Medeiros Lorentz TC, Cortelli SC, Cortelli JR (2013). Progression of periodontitis and tooth loss associated with glycemic control in individuals undergoing periodontal maintenance therapy: a 5-year follow-up study. J Periodontol.

[72] Zhu Y, Hollis JH (2015). Associations between the number of natural teeth and metabolic syndrome in adults. J Clin Periodontol.

[73] Hyvarinen K, Salminen A, Salomaa V, Pussinen PJ (2015). Systemic exposure to a common periodontal pathogen and missing teeth are associated with metabolic syndrome. Acta Diabetol.

[74] Kim S-W, Cho K-H, Han K-D, Roh Y-K, Song I-S, Kim Y-H (2016). Tooth loss and metabolic syndrome in South Korea: the 2012 Korean National Health and nutrition examination survey. Medicine (Baltimore).

[75] Graziani F, Gennai S, Solini A, Petrini M (2018). A systematic review and meta-analysis of epidemiologic observational evidence on the effect of periodontitis on diabetes an update of the EFP-AAP review. J Clin Periodontol.

[76] Noma H, Sakamoto I, Mochizuki H, Tsukamoto H, Minamoto A, Funatsu H, Yamashita H, Nakamura S, Kiriyama K, Kurihara H, Mishima HK (2004). Relationship between periodontal disease and diabetic retinopathy. Diabetes Care.

[77] Aoyama N, Suzuki J-I, Kobayashi N, Hanatani T, Ashigaki N, Yoshida A, Shiheido Y, Sato H, Minabe M, Izumi Y, Isobe M (2018). Associations among tooth loss, systemic inflammation and antibody titers to periodontal pathogens in Japanese patients with cardiovascular disease. J Periodontal Res.

[78] Joshy G, Arora M, Korda RJ, Chalmers J, Banks E (2016). Is poor oral health a risk marker for incident cardiovascular disease hospitalisation and all-cause mortality? Findings from 172 630 participants from the prospective 45 and up study. BMJ Open.

[79] Li Q, Chalmers J, Czernichow S, Neal B, Taylor BA, Zoungas S (2010). Oral disease and subsequent cardiovascular disease in people with type 2 diabetes: a prospective cohort study based on the action in diabetes and vascular disease: Preterax and Diamicron modified-release controlled evaluation (ADVANCE) trial. Diabetologia.

[80] Sandberg GE, Wikblad KF (2003). Oral health and health-related quality of life in type 2 diabetic patients and non-diabetic controls. Acta Odontol Scand.

[81] Association AD (2018). Economic costs of diabetes in the U.S. in 2017. Diabetes Care.

[82] Zhu Y, Hollis JH (2014). Tooth loss and its association with dietary intake and diet quality in American adults. J Dent.

[83] Hashemi R, Rahimlou M, Baghdadian S, Manafi M (2019). Investigating the effect of DASH diet on blood pressure of patients with type 2 diabetes and prehypertension: randomized clinical trial. Diabetes Metab Syndr.

[84] Working Together to Manage Diabetes: A Toolkit for Pharmacy, Podiatry, Optometry, and Dentistry (PPOD) | Toolkits | NDEP | Diabetes | CDC 2019.

[85] Diabetes and You: Healthy Teeth Matter! n.d. https://www.cdc.gov/diabetes/ndep/pdfs/150-healthy-teeth-matter.pdf.

